# Peer Review of “Left Ventricular Outflow Tract Obstruction in Patients Treated With Milrinone for Cerebral Vasospasm: Case Report and Literature Review”

**DOI:** 10.2196/37056

**Published:** 2022-04-11

**Authors:** Uday Kumar Chalwadi

**Affiliations:** 1 Clinical Trials Innovation Unit Translational Research Institute University of Arkansas for Medical Sciences Little Rock, AR United States

**Keywords:** ventricular outflow obstruction, subarachnoid hemorrhage, vasospasm, intracranial, milrinone, hemorrhage, neurosurgery, neurology, surgery, pharmaceutical

*This is a peer-review report submitted for the paper “Left Ventricular Outflow Tract Obstruction in Patients Treated With Milrinone for Cerebral Vasospasm: Case Report and Literature Review.”*

## Review Round 1

### Reviewer AA

#### General Comments

This is an interesting paper [1]. Overall, the information is well presented. That said, there are some areas that need improvements.

#### Specific Comments

##### Major Comments

1. This type of left ventricular outflow tract obstruction (LVOTO) should be addressed as dynamic LVOTO.
2. LVOTO per se should be briefly explained in the “Introduction” for the benefit of noncardiology readers: what LVOTO means, types of LVOTO such as fixed and dynamic, and a brief and simple explanation of dynamic LVOTO.
3. For the second patient, pages 7 and 8 state “In view of the hemodynamic improvement and the good neurological course, treatment with milrinone was continued at the same dose.” It looks like a repeat echo was done only after stopping milrinone. Was any echo repeated after hemodynamic improvement while the patient was continued on milrinone? How did you come to the conclusion that LVOTO is because of milrinone? He also had meningitis/sepsislike state (mentioned as an inflammatory syndrome in the manuscript), which in itself could predispose to LVOTO. Additionally, LVOTO can occur postoperatively after noncardiac surgery in patients with no known heart disease, and this patient also had a surgical procedure in the form of ventricular drain. These aspects are well discussed in reference 16 of the manuscript.
4. What is the explanation for unilateral left sided pulmonary edema for the first patient (as pulmonary edema is mostly bilateral in heart failure).

##### Minor Comments

1. The authors mention vasospasm was diagnosed using a computerized tomography (CT) scan. Plain CT scans are not used for the diagnosis of vasospasm, and they need to be more specific as to how vasospasm was diagnosed (eg, CT angio, Doppler study, or perfusion scan).

## Review Round 2

### Specific Comments

#### Major Comments

1. Page 6, lines 10-12 states “Although milrinone was administered at a constant dosage of 1 μg/kg/min, the clinical presentation led to find the origin of the shock: an accidental bolus of a milrinone due to a plication of the central venous catheter line during nursing care”. Would recommend clarifying this statement and explaining what exactly you mean by plication and how it resulted in an accidental bolus of milrinone.
2. Bedside limited echocardiography is a routine practice to check the effect of various interventions in the intensive care unit. Therefore, it should be explained why echocardiography was not repeated in the second patient after hemodynamic improvement while the patient was continued on milrinone. Just relying on “systolic murmur” is not enough. Moreover, a murmur is also not described in detail. The murmur description should include intensity, quality, radiation, timing (pan systolic/short systolic), etc.
3. “Mitral regurgitation associated with LVOTO is most often eccentric, and travels to the left pulmonary veins, resulting in unilateral acute pulmonary edema in this patient.” Please provide a reference for this.

## Abbreviations

CT: computerized tomography
LVOTO: Left Ventricular Outflow Tract Obstruction
